# The Use of Point of Care Ultrasound in Diagnosis of Peritonsillar Abscess

**DOI:** 10.24908/pocus.v8i2.16568

**Published:** 2023-11-27

**Authors:** Brian Kohen, Melanie Perez, Jheanelle Mckay, Rolando Zamora, Curtis Xu

**Affiliations:** 1 Memorial Healthcare System Pembroke Pines, Florida USA; 2 University of Texas Southwestern Medical Center Dallas, Texas USA; 3 Joe DiMaggio's Children's Hospital Hollywood, Florida USA; 4 University of Connecticut Health Center Farmington, Connecticut USA

**Keywords:** POCUS, Emergency Ultrasound, Peritonsillar Abscess, ENT, Pediatrics Case Presentation

## Abstract

The use of point of care ultrasound (POCUS) for diagnosis and treatment of peritonsillar abscess (PTA) is increasing 1. Proven advantages include improved diagnostic accuracy and treatment success rates as well as decreased otolaryngology consultation, computed tomography (CT) usage, return visits to the emergency department (ED), and length of stay 1. We present a case of a patient with a PTA that was diagnosed and successfully treated utilizing POCUS, avoiding the need for otolaryngology consultation and CT.

## Case Presentation

**Figure 1:**
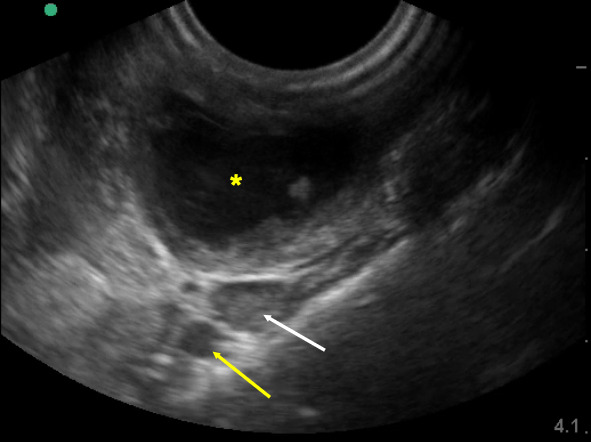
Peritonsillar abscess (*) adjacent to the internal carotid artery (yellow arrow) and internal jugular vein (white arrow) at a depth of about 3cm .

An 18-year-old girl who recently recovered from infectious mononucleosis presented to the pediatric emergency department (ED) with a sore throat. She had completed a course of steroids due to persistent sore throat associated with right-sided neck pain and hoarseness. Over the following 2 days, her symptoms worsened and progressed to odynophagia and trismus, prompting her visit to the ED. In the ED, she was well appearing in no acute distress with normal vital signs. On examination, she had a leftward uvular deviation in addition to a mass in the right peritonsillar space, concerning for a PTA. An intraoral POCUS was performed using an endocavitary probe to better visualize the abscess and plan for drainage (Video S1). Using POCUS, the emergency physicians were able to confirm the abscess, measure its size, and identify the depth of nearby vessels to avoid while performing the drainage (Figure 1). The PTA was subsequently safely and successfully drained, yielding about 7 milliliters of pus which later grew Streptococcus pyogenes. The patient experienced immediate relief and was discharged on oral antibiotics.

## Discussion

PTA is a common deep neck space infection, with an incidence of about 3 in 10,000 per year 2. Physical examination is often unreliable, with reported sensitivity and specificity of 78% and 50%, respectively, even amongst experienced specialists 3. Moreover, it is often difficult to distinguish between peritonsillar abscess and other deep neck space infections such as tonsillitis and peritonsillar cellulitis clinically 4. Imaging modalities such as CT are accurate but have several disadvantages such as increased costs, length of stay, intravenous contrast and radiation risk. POCUS has increasingly been used as an adjunct for diagnosis of PTA, with a sensitivity ranging from 89-95% and specificity of 78-100% 3, 5. Additionally, when used for the treatment of PTA, POCUS increases successful drainage and diagnostic accuracy, and decreases otolaryngology consultation, CT utilization, return visits to the ED, and length of stay 1, 6, 7. In our case, PTA was rapidly diagnosed and successfully treated in the ED without the need for otolaryngology consultation or CT.

## Disclosures

The authors report no disclosures related to this work. 

## Supplementary Material

 Video S1 Discrete peritonsillar fluid collection diagnostic of a peritonsillar abscess.
